# First- and Second-Order Full-Differential in Edge Analysis of Images

**DOI:** 10.1155/2014/121928

**Published:** 2014-06-25

**Authors:** Dong-Mei Pu, Yu-Bo Yuan

**Affiliations:** School of Science, China Jiliang University, Hangzhou 310018, China

## Abstract

Two concepts of first- and second-order differential of images are presented to deal with the changes of pixels. These are the basic ideas in mathematics. We propose and reformulate them with a uniform definition framework. Based on our observation and analysis with the difference, we propose an algorithm to detect the edge from image. Experiments on Corel5K and PASCAL VOC 2007 are done to show the difference between the first order and the second order. After comparison with Canny operator and the proposed first-order differential, the main result is that the second-order differential has the better performance in analysis of changes of the context of images with good selection of control parameter.

## 1. Introduction

In 1920s, digital image edge detection is becoming an important technology in image processing. With the development of electronic technology, computer technology and communication technology, edge detection of digital image had become a hot issue with the high-speed development. After decades of development, edge detection of digital image processing technology has been widely applied in many important fields of industrial, microbial field, medicine, aerospace, and defense; extensive attention has been a technology powerhouse of the world [1].

The development of processing technology of digital image edge detection in the recent 10 years is rapid. Many algorithms are published in each year after 1990s. Among of them, Canny, wavelet transform and log algorithms had important influence in edge detection. The same ideas are extracted from relevant knowledge of mathematics. These ideas also had been used in many important fields, such as digital signal processing, information theory and chroma and so on. Many other ideas are form neural network, genetic algorithm, artificial intelligence and fuzzy logic theory and so on [1–12].

Modern digital image edge detection has three objectives: visualization, automation, and quantitative.

- Visualization: when the images are acquired and displayed, the image usually needs to be improved in order to be easier to the observer to explain them. The target of interest to highlight or image parts needs contrast enhancement. Since the advent of science, techniques like CT and MRI 3D imaging technique of visualization, especially the visualization of 3D structure, have attracted great attention.
- Automation: it aims to make some daily or tedious work automation. For example, according to the automatic determination system of chromosome karyotype of a chromosome distribution in the image, the system automatically generates differential leukocyte count report from a blood smear. Characteristics of these applications are the minimum human intervention, automatic complete the analysis work.By application of differential leukocyte count, commercially available systems are developed in the 1970s. But today, this task is carried out in a totally different way (by flow cytometry leukocyte count technology), that is, automatically.
- Quantitative: image edge is detected by quantitative examples, procedures for the measurement of arterial stenosis, and observing the localization and quantitation of special components in tissue sections with electron microscope (such as hemochromatosis, iron). In these applications, the aim is to allow human intervention because the processing time in these applications is not the principal contradiction.

Edge detection in digital image processing carries on processing to the image edge with computer; this technology is a new field of application with the development of computer technology and opened up many aspects of the optics, electronics, mathematics convergence, photography, computer technology, and other disciplines. Edge detection of image processing as a discipline has been American Mathematical Society as a branch of applied mathematics. In its short development history, it has been successfully applied in almost all domains related to the formation of image. In recent years, image analysis and processing, realize tightly around the theory, application of three aspects of rapid development. It takes many subjects as the theoretical basis; it penetrated into many disciplines and became the theory practice, new branch occupying an important position in the field of high technology.

## 2. Concepts of Differential and of Total Differential of Images

From the views of human and machine visions, basically, we get the objection of image in *R* ^2^. Based on the fact, we redefined one image as an observation on the domain [*a*, *b*]×[*c*, *d*] ∈ *R* ^2^. In discrete spaces of *R* ^2^, one color image is denoted by matrix

(1) $$\begin{array}{c} I=\left(\begin{array}{cccc} P_{11} & P_{12} & \cdots & P_{1n}\\ P_{21} & P_{22} & \cdots & P_{2n}\\ \cdots & \cdots & \cdots \cdots & \cdots \\ P_{m1} & P_{m2} & \cdots & P_{mn} \end{array}\right), \end{array}$$

where **P** is a pixel in color space of  *R* ^3^ with elements* red*,* green*, and* blue*.

(2) $$\begin{array}{c} P=\left(\begin{array}{ccc} P^{R} & P^{G} & P^{b} \end{array}\right). \end{array}$$

**Definition 1 .**

**Figure 1:**
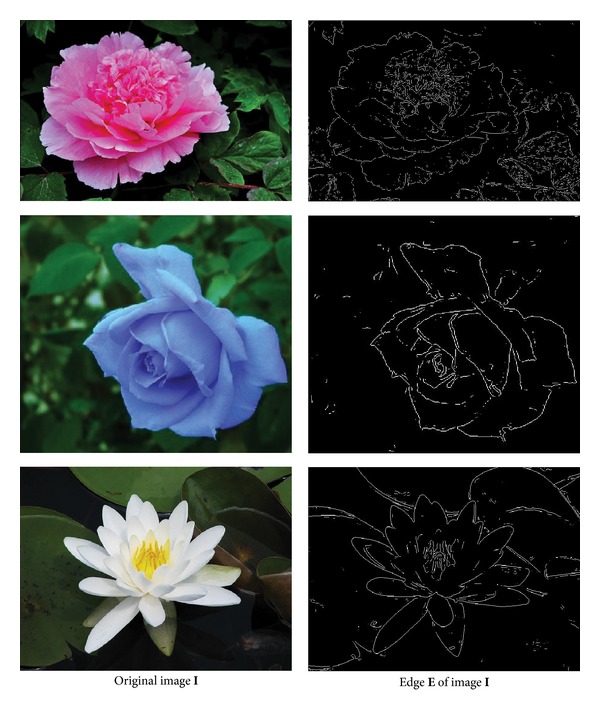
Illustration of relation between image and edge.

In the discrete space, edge **E** ∈ *R* ^*m*×*n*^ of image **I** ∈ *R* ^*m*×*n*^ is defined as the discontinuous image characteristics. In practice, it refers to the local image characteristics such as mutation, gray level, color change, and texture change. Edge widely exists in the target and objective, foreground and background, salient region and un-salient ones. It is an important feature in image segmentation, some compared images between images and their edges can be seen in Figure 1.

Mathematically, the basic idea of edge is the difference of neighbor pixels or the change of two points. In other words, the edge has very important relation with first- and second-order differential operators.

**Definition 2 .**

Let **I**(*x*, *y*) be a continuous function in [*a*, *b*]×[*c*, *d*], given (*x*_0_, *y*_0_)∈(*a*, *b*)×(*c*, *d*),  (*x*_0_ + Δ*x*, *y*_0_ + Δ*y*)∈(*a*, *b*)×(*c*, *d*), the first difference is

(3) ΔI=I(x0+Δx,y0+Δy)−I(x0,y0+Δy)+I(x0,y0+Δy)−I(x0,y0).

If

(4) $$\begin{array}{c} \Delta I=G\left(x_{0},y_{0}\right)\left(\begin{array}{c} \Delta x\\ \Delta y \end{array}\right)+o\left(\sqrt{{\left(\Delta x\right)}^{2}+{\left(\Delta y\right)}^{2}}\right), \end{array}$$

then we call that **I**(*x*, *y*) is differentiable at point (*x*_0_, *y*_0_);

(5) $$\begin{array}{c} G\left(x_{0},y_{0}\right)\left(\begin{array}{c} \Delta x\\ \Delta y \end{array}\right) \end{array}$$

is the differential of **I**(*x*, *y*) at point (*x*_0_, *y*_0_). We denote it as

(6) $$\begin{array}{c} dI=G\left(x_{0},y_{0}\right)\left(\begin{array}{c} \Delta x\\ \Delta y \end{array}\right). \end{array}$$

In general,

(7) $$\begin{array}{c} dI=A\left(x_{0},y_{0}\right)\Delta x+B\left(x_{0},y_{0}\right)\Delta y. \end{array}$$

Also, *G*(*x*_0_, *y*_0_) is called the gradient of the image **I**(*x*, *y*) at pixel (*x*_0_, *y*_0_), and

(8) $$\begin{array}{c} A\left(x_{0},y_{0}\right)={\frac{\partial I}{\partial x}|}_{\left(x_{0},y_{0}\right)},\;\;B\left(x_{0},y_{0}\right)={\frac{\partial I}{\partial y}|}_{\left(x_{0},y_{0}\right)}. \end{array}$$

**Definition 3 .**

The edge **E** with the first-order difference is defined as

(9) E(x0,y0) ={1,if  ||dI(x0,y0)||>c1,  (x0,y0)∈(a,b)×(c,d);0,if  ||dI(x0,y0)||≤c1,  (x0,y0)∈(a,b)×(c,d),

where ||·|| is somewhat norm which satisfied the basic rules of norm definition and *c*_1_ is the control parameter to determine edge points.

**Definition 4 .**

Let **I**(*x*, *y*) be a differentiable function in (*a*, *b*)×(*c*, *d*), given (*x*_0_, *y*_0_)∈(*a*, *b*)×(*c*, *d*),  (*x*_0_ + Δ*x*, *y*_0_ + Δ*y*)∈(*a*, *b*)×(*c*, *d*), the second difference is

(10) Δ2I=ΔI(x0+Δx,y0+Δy)−ΔI(x0,y0+Δy)+ΔI(x0,y0+Δy)−ΔI(x0,y0).

If

(11) $$\begin{array}{c} \Delta^{2}I=H\left(x_{0},y_{0}\right)\left(\begin{array}{c} \Delta x\\ \Delta y \end{array}\right)+o\left(\sqrt{{\left(\Delta x\right)}^{2}+{\left(\Delta y\right)}^{2}}\right), \end{array}$$

then we call that **I**(*x*, *y*) is second differential at point (*x*_0_, *y*_0_);

(12) $$\begin{array}{c} H\left(x_{0},y_{0}\right)\left(\begin{array}{c} \Delta x\\ \Delta y \end{array}\right) \end{array}$$

is the differential of **I**(*x*, *y*) at point (*x*_0_, *y*_0_). We denote it as

(13) $$\begin{array}{c} d^{2}I=H\left(x_{0},y_{0}\right)\left(\begin{array}{c} \Delta x\\ \Delta y \end{array}\right). \end{array}$$

In general,

(14) $$\begin{array}{c} H\left(x_{0},y_{0}\right)={\left(\begin{array}{cc} \frac{\partial^{2}I}{\partial x\partial x} & \frac{\partial^{2}I}{\partial x\partial y}\\ \frac{\partial^{2}I}{\partial y\partial x} & \frac{\partial^{2}I}{\partial y\partial y} \end{array}\right)|}_{\left(x_{0},y_{0}\right)}. \end{array}$$

**Definition 5 .**

Let **I**(*x*, *y*) be a twice differentiable function in (*a*, *b*)×(*c*, *d*), given (*x*_0_, *y*_0_)∈(*a*, *b*)×(*c*, *d*), the twice differential of  **I**(*x*, *y*) at point (*x*_0_, *y*_0_) is

(15) $$\begin{array}{c} d^{2}I\left(x_{0},y_{0}\right)=\left(\begin{array}{cc} \Delta x & \Delta y \end{array}\right)H\left(x_{0},y_{0}\right)\left(\begin{array}{c} \Delta x\\ \Delta y \end{array}\right). \end{array}$$

**Definition 6 .**

The edge **E** with the first-order difference is defined as

(16) E(x0,y0) ={1,if  ||d2I(x0,y0)||>c2,  (x0,y0)∈(a,b)×(c,d);0,if  ||d2I(x0,y0)||≤c2,  (x0,y0)∈(a,b)×(c,d),

where ||·|| is somewhat matrix norm which satisfied the basic rules of matrix norm definition and *c*_2_ is also the control parameter to determine edge points.

## 3. Algorithms

See Algorithm 1.

## 4. Experiments

In order to make our experiment convincing, we choose our experiment data from the well-known image datasets Corel5K and Pascal VOC 2007, which are widely used in image annotation.

**Figure 2:**
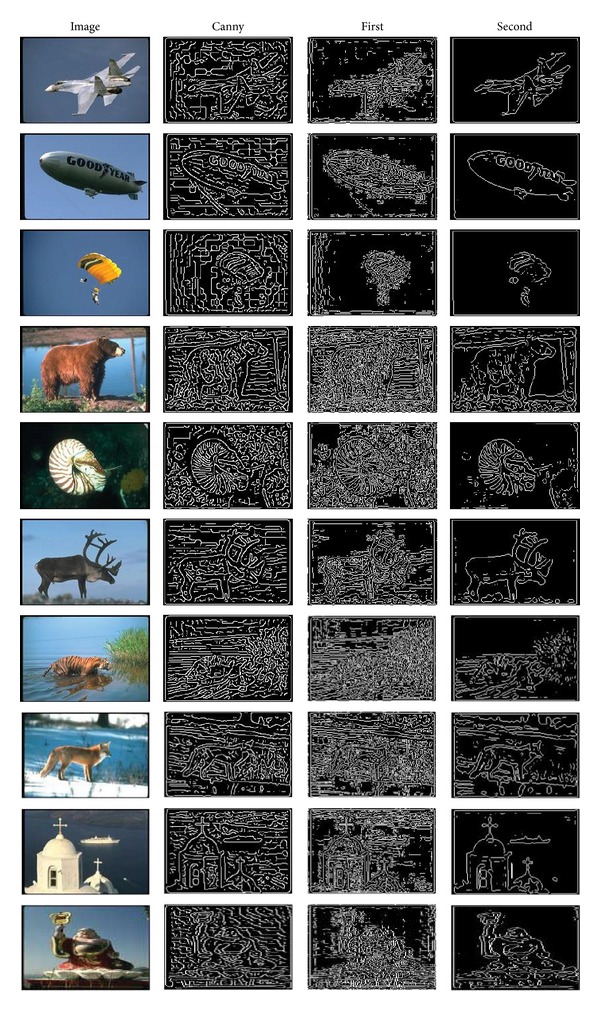
Edge detection results with first and second differential.

We selected some original pictures from these two databases. They are shown in Figure 2. The Corel5K image corpus is a publicly available and widely used dataset in evaluating image annotation methods. It contains 5000 images from 50 themes with 100 images from each theme. Pascal VOC Challenge is a famous competition in computer vision which is run each year since 2005; the goal of this challenge is to recognize objects from a number of visual object classes in realistic scenes.

We employ Canny operator (a well-known edge detector) as the baseline. After employing the algorithms, we can obtain the conclusion that the twice-order differential is more effective to detect the edge from the given data sets of images. Some excellent results can be shown in Figure 2.

## 5. Conclusions

The concepts of first- and second-order differential of images are proposed to deal with the changes of pixels. A uniform definition framework of them is presented. After careful observation and analysis of the difference, we employ them to study the edge detection of images. Experiments on Corel5K and PASCAL VOC 2007 can show the difference between the first order and the second order. The important is that the second-order differential has the better performance in analysis of changes of the context of images.

## Figures and Tables

**Algorithm 1:**
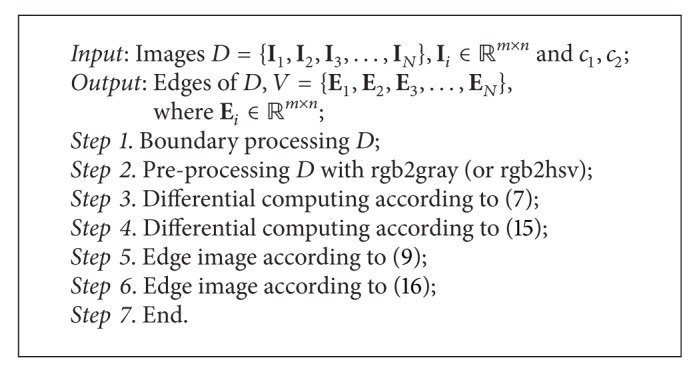
Edge Detection with First and Second Differential.
